# Histidine-improved cancer immunosurveillance

**DOI:** 10.1080/2162402X.2026.2713805

**Published:** 2026-08-13

**Authors:** Marine Fidelle, Ella Reich, Laurence Zitvogel, Guido Kroemer

**Affiliations:** a Université Paris-Saclay, Gustave Roussy, ClinicObiome, Inserm UMR1367, Microbiota and Mucosal Immunity for Cancer Immunotherapy, Villejuif, France; b Centre de Recherche des Cordeliers, Équipe labellisée par la Ligue contre le cancer, Inserm U1138, Université Paris Cité, Sorbonne Université, Paris, France; c Metabolomics and Cell Biology Platforms, UMS AMMICa, Gustave Roussy Institut , Villejuif, France; d Institut du Cancer Paris CARPEM, Department of Biology, Hôpital Européen Georges Pompidou, AP-HP, Paris, France

Cancer immunotherapy has transformed clinical oncology, yet durable responses to immune checkpoint inhibitors (ICI) remain restricted to a minority of patients. Although genomic and immunological determinants of response have been intensively investigated, it is becoming increasingly clear that systemic metabolism and host-microbiota interactions critically shape anticancer immunosurveillance. In this context, our recent work published in *Nature Medicine* provides compelling evidence that circulating histidine may constitute both a biomarker and a metabolic effector of successful immunotherapy.^1^ Using longitudinal plasma metabolomics monitoring 154 metabolites in more than 1,700 patients in 16 cohorts from eight European, UK and North American countries across 5 tumor types and multiple therapeutic settings, we identified histidine as one of the strongest positive predictors of progression-free survival during immunotherapy. Importantly, this observation was reproduced across seven independent validation cohorts and corroborated by mechanistic studies in mouse tumor models. Oral histidine supplementation enhanced the efficacy of PD-1 or CTLA-4 blockade, increased pre-effector CD8^+^ T-cell fitness, stimulated mitochondrial fatty acid oxidation and reduced exhaustion-associated dysfunction.^1^

Altogether, these findings fit well within the emerging concept that “metabolic checkpoints” govern the amplitude and durability of immune responses against cancer. Metabolites are not merely passive bystanders reflecting nutritional status or tumor burden; rather, they directly modulate T-cell differentiation, dendritic-cell function, macrophage polarization and immunological memory.^2^

Histidine is particularly intriguing because it occupies a nodal position linking amino acid availability, one-carbon metabolism, mitochondrial fitness and microbial biotransformation. Histidine degradation feeds into folate-dependent one-carbon metabolism through formiminotetrahydrofolate intermediates, potentially supporting nucleotide biosynthesis and mitochondrial respiration in activated lymphocytes.^3^ This may be especially relevant in aged or chronically stimulated T cells, which progressively lose metabolic flexibility.^4^ Indeed, in mice, histidine supplementation can directly enhance T-cell bioenergetics. Histidine-exposed CD8^+^ T cells displayed improved oxidative metabolism and preserved proliferative potential under repetitive stimulation conditions compatible with exhaustion (Figure 1).^1^ Such observations resonate with prior demonstrations that mitochondrial fitness and fatty acid oxidation are indispensable for sustained T-cell effector function during chronic antigen exposure.^5^

**Figure 1.**
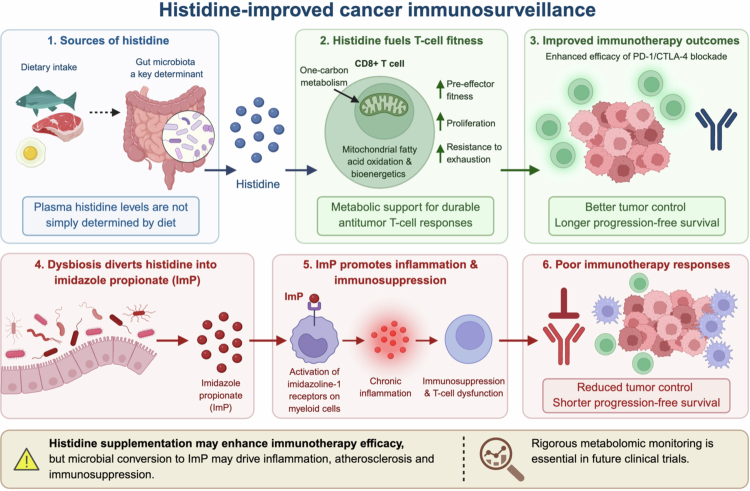
Histidine-improved cancer immunosurveillance. Histidine acts as a systemic metabolic determinant of anticancer immunity and immunotherapy efficacy. Plasma histidine levels are influenced by both diet and the gut microbiota. Increased histidine availability supports CD8⁺ T-cell fitness by enhancing one-carbon metabolism, mitochondrial function and fatty-acid oxidation, thereby promoting pre-effector differentiation, proliferation, and resistance to exhaustion. These metabolic effects improve the efficacy of immune checkpoint blockade (PD-1 and CTLA-4 inhibitors), resulting in superior tumor control and prolonged progression-free survival (boxes 1, 2, 3 referring to Suissa et al. Nat. Med. 2026). Conversely, dysbiotic microbiota can divert histidine metabolism toward the production of imidazole propionate (ImP), a microbial metabolite associated with metabolic inflammation and cardiovascular disease. ImP activates inflammatory pathways in myeloid cells, leading to chronic inflammation, immunosuppression and T-cell dysfunction. Excessive ImP generation may therefore counteract the beneficial effects of histidine and may contribute to poor responses to immunotherapy (boxes 4, 5, 6). The scheme highlights the dual nature of histidine metabolism: while histidine supplementation may enhance anticancer immunosurveillance, its microbial conversion into ImP may promote inflammation, atherosclerosis and immune dysfunction. These observations underscore the need for microbiome profiling and metabolomic monitoring in future clinical trials evaluating histidine-based metabolic interventions. Figure created in BioRender. 1015, L. (2026) https://BioRender.com/i7uxdvb.

Another important aspect of this work concerns the intimate interplay between dietary histidine and the intestinal microbiota. Although histidine is classified as an essential amino acid, plasma histidine concentrations did not simply reflect dietary intake of fish, meat or eggs^1^ in line with previous observations that circulating histidine concentrations are similar in vegan as in omnivorous individuals.^6^ ^,^ ^7^ Instead, gut microbial ecology emerged as a major determinant of histidine availability and downstream metabolism.^1^ This observation is conceptually important because it underscores that humans should be viewed as metaorganisms in which microbial metabolism profoundly shapes systemic immunological tone including through the neosynthesis, as well as the avoidance of the degradation of essential amino acids such as histidine. Particularly noteworthy is the role of imidazole propionate (ImP), a microbial histidine-derived metabolite that emerged as a potential antagonist of beneficial histidine signaling. In dysbiotic microbiota configurations, fecal and plasma ImP levels increased, while favorable responses to immunotherapy declined (Figure 1).^1^

This latter observation is highly significant because accumulating evidence indicates that ImP is not a neutral catabolic byproduct but rather a biologically active metabolite with deleterious systemic effects. Indeed, ImP has already been implicated in metabolic inflammation and cardiometabolic disease including diabetes, heart failure and atherosclerosis.^8-10^ At least part of this deleterious effect is mediated through the action of ImP on imidazoline-1 receptors expressed by myeloid cells.^10^

The implications for oncology may be profound. Chronic inflammation does not necessarily translate into productive antitumor immunity. On the contrary, persistent inflammatory signaling frequently drives myelopoiesis, T-cell dysfunction, macrophage polarization and immune suppression. Thus, ImP may represent a paradoxical “dark side” of histidine metabolism, converting an immunostimulatory nutrient into an immunosuppressive and potentially pro-atherogenic mediator.^8-10^ This possibility deserves serious consideration because many cancer patients receiving immunotherapy present with metabolic syndrome, systemic inflammation and cardiovascular comorbidities.^11^ In this context, indiscriminate oral histidine supplementation without metabolic monitoring could theoretically exacerbate inflammatory or cardiovascular complications in subsets of patients harboring unfavorable microbiota compositions.

Accordingly, future clinical studies evaluating histidine supplementation should not merely administer histidine as a nutritional adjunct but instead incorporate rigorous companion biomarker strategies. Longitudinal plasma and fecal metabolomics should systematically quantify histidine, histamine, urocanate derivatives and especially ImP. Simultaneously, shotgun metagenomics or validated dysbiosis signatures should identify microbial ecosystems capable of excessive histidine degradation toward ImP production. Such precision monitoring will likely be indispensable to identify patients in whom histidine supplementation enhances immunosurveillance *versus* those in whom it may fuel pathogenic inflammatory pathways.

An additional concern relates to histamine, another major downstream product of histidine catabolism. Histamine signaling through H1–H4 receptors can exert immunosuppressive effects within tumors and may contribute to T-cell dysfunction.^12^ ^,^ ^13^ Although we did not detect circulating histamine in their cohorts, local tissue production may nonetheless remain biologically relevant.^1^

Pending further exploration, the current data do not support simplistic nutritional recommendations. Histidine should not yet be viewed as a universal “immune-boosting” supplement for cancer patients. Rather, histidine metabolism appears highly context-dependent, shaped by microbial ecology, inflammatory status, mitochondrial competence and host metabolic reserve. The same metabolic pathway may either support productive anticancer immunity or generate inflammatory metabolites associated with immunosuppression and vascular pathology.

In spite of these caveats, the translational potential remains substantial. Histidine supplementation appears relatively safe in humans, and the preclinical evidence supporting immunostimulatory activity is persuasive. Carefully designed interventional studies integrating microbiome stratification and metabolomic surveillance could therefore open a new avenue for metabolic immunotherapy.

The broader conceptual advance is perhaps even more important than histidine itself. Our recent work reinforces the emerging paradigm that successful cancer immunotherapy may require simultaneous manipulation of immune checkpoints, microbial ecology, and systemic metabolism. Histidine now joins a growing list of metabolites capable of influencing cancer immunosurveillance, together with inosine, arginine, succinate, kynurenines, short-chain fatty acids, polyamines, and bile acid derivatives.^2^

Future oncological interventions may thus evolve toward integrated “immunometabolic ecosystems” in which nutrition, microbial engineering and immunotherapy are therapeutically coordinated. Within this framework, histidine represents both an opportunity and a warning: metabolic interventions can stimulate anticancer immunity, yet their downstream microbial transformation products may simultaneously impose inflammatory and immunosuppressive liabilities. Understanding and controlling this duality will be essential for the safe clinical translation of histidine-based immunotherapeutic strategies.

## Data Availability

This paper does not contain any original data.
